# Managing the Morbidity Associated with Respiratory Viral Infections in Children with Congenital Heart Disease

**DOI:** 10.1155/2012/646780

**Published:** 2012-02-29

**Authors:** Joseph M. Geskey, Stephen E. Cyran

**Affiliations:** ^1^Division of Pediatric Hospital Medicine, Penn State College of Medicine, 500 University Drive, P.O. Box 850, Hershey, PA 17033, USA; ^2^Division of Pediatric Cardiology, Penn State College of Medicine, Hershey, PA 17033, USA

## Abstract

Children with congenital heart disease (CHD) are at risk for increased morbidity from viral lower respiratory tract infections because of anatomical cardiac lesions than can worsen an already compromised respiratory status. Respiratory syncytial virus (RSV) remains an important pathogen in contributing toward the morbidity in this population. Although the acute treatment of RSV largely remains supportive, the development of monoclonal antibodies, such as palivuzumab, has reduced the RSV-related hospitalization rate in children with CHD. This review highlights the specific cardiac complications of RSV infection, the acute treatment of bronchiolitis in patients with CHD, and the search for new therapies against RSV, including an effective vaccine, because of the high cost associated with immunoprophylaxis and its lack of reducing RSV-related mortality.

## 1. Introduction

Surgical outcomes in children with congenital heart disease (CHD) have improved over the past two decades because of advances in prenatal diagnosis, surgical and cardiopulmonary bypass techniques, and postoperative management [1]. In fact, it is expected that adult patients with CHD now exceed the number of children with CHD [2], and the majority of patients who have undergone surgical correction for their CHD do not consider their quality of life to be impacted by their malformation [3].

 These improved outcomes require a significant economic investment so that processes of care are optimally aligned throughout the continuum of a patient's life from initial diagnosis, through his or her surgical correction (including postoperative management) and after hospitalization follow-up. However, a significant number of these children will require multiple surgeries before they are fully corrected and, therefore, can be exposed to community-acquired respiratory infections which can result in substantial morbidity and mortality. Outside the perioperative period, respiratory infections are significantly more likely to be the cause of death during the winter than the summer [4]. Consequently, it is critical for both primary care physicians and pediatric cardiologists to remain familiar with the current epidemiology and clinical management of respiratory pathogens in children with CHD, while also understanding what contemporary prophylactic options exist in minimizing the burden of viral respiratory infections throughout this population.

 Over one-half of hospital admissions related to acute respiratory tract infections in children with CHD are due to bronchiolitis [5]. In the United States, the number of outpatient visits and the number and percentage of yearly hospitalizations for bronchiolitis have actually increased since 1980 [6, 7]. Respiratory syncytial virus (RSV) remains the most common etiologic agent of bronchiolitis and is responsible for up to 90% of hospitalizations due to bronchiolitis [8]. Children with CHD have higher rates of hospitalization, a longer length of stay, and risk of death if they become infected with RSV compared to children without CHD [9–11].

It is estimated that there are 33.8 million episodes of RSV lower respiratory tract infection (LRTI) worldwide, with children in developing countries twice as likely to be infected as compared to children in industrial countries. Approximately 10% of these episodes will lead to hospitalization, and it is estimated that 66,000–199,000 children will die from RSV-associated LRTI; 99% of whom will be from developing countries [12].

## 2. Respiratory Syncytial Virus

 RSV is an enveloped RNA virus whose genome encodes several structural proteins including the (G) protein, which promotes viral attachment, and the fusion (F) protein, which promotes penetration. There are two groups, A and B, of RSV strains that circulate together during yearly outbreaks [8].

### 2.1. Clinical Symptoms and Diagnosis

 Typically patients have rhinorrhea and a low-grade fever that can progress to cough and respiratory distress over the next few days and can impair feeding and sleeping. Infants <3 months of age can also present with apnea [13]. Patients typically will have crackles and/or wheezes on lung exam and depending on the severity of infection, tachypnea, chest wall retractions, and hypoxia. The diagnosis is typically made clinically based on the above findings, although viral culture, direct fluorescent assays, and rapid antigen detection can be used. Also, the development of real-time polymerase chain reaction (RT-PCR) testing has allowed more rapid detection of respiratory pathogens in the setting of pediatric respiratory infections.

### 2.2. The Impact of Viral Respiratory Infections on CHD

 Cabalka [14] highlighted the physiologic risk factors in children with CHD that can contribute to worse clinical outcomes from viral respiratory infections. Children who have left-to-right shunt lesions may have increased pulmonary blood flow that leads to pulmonary edema and decreased functional residual capacity. These alterations in pulmonary mechanics further lead to areas of atelectasis and ventilation-perfusion mismatch, ultimately leading to hypoxia. Patients with cyanotic congenital heart disease may experience intensification in their baseline level of cyanosis due to bronchiolitis-related reductions in lung volume and airway diameter superimposed on their already reduced pulmonary blood flow and obligatory right-to-left shunt. Children with CHD may also have impaired ventricular function leading to higher pulmonary venous pressures, capillary leak, and pulmonary edema which further contribute to increased ventilation-perfusion mismatch.

 Although earlier reports indicated mortality rates of 37% for RSV-infected CHD patients and 73% in patients with pulmonary hypertension [15], more recent studies suggest that mortality rates have decreased to <2%, even in children with pulmonary hypertension [16]. Improvements in critical care medicine have led to the decrease in mortality rates but screening for RSV prior to surgical intervention also has contributed to a reduction in RSV-related pulmonary hypertension and therefore a reduction in postoperative morbidity [17, 18]. Risk factors for RSV hospitalization in children with heart disease include trisomy-21, cardiomyopathy, and hemodynamically significant heart disease. Younger patients, particularly those with heart failure, can be expected to have a more severe clinical course [19]. 

 Other viruses other than RSV are responsible for bronchiolitis. The development of molecular testing for viruses obtained from nasopharyngeal swabs or aspirates via RT-PCR has highlighted the role of dual infections in patients with viral respiratory tract infections. However, the role of dual infection correlating to increased clinical severity is unclear. Although some investigators have reported no clinical differences in patients with dual infections [20–22], others have reported more severe bronchiolitis in patients who have had multiple viruses detected [23–26]. In the studies that demonstrated a more severe clinical infection with dual respiratory pathogens, two studies specifically excluded children with CHD [23, 25] while the other two studies enrolled children with CHD but did not separately report clinical outcomes in this group [24, 26]. Richard et al. [26], however, did include children with CHD as part of a cohort of children with underlying chronic illness and reported that dual infection with RSV and rhinovirus was significantly associated with admission to the Pediatric Intensive Care Unit. There is also no evidence that the availability to diagnose dual pathogens via RT-PCR testing leads to shorter hospital stays or reduces antibiotic use in children with acute respiratory infection [27]. 

 There are different risk factors between RSV and non-RSV bronchiolitis. In one study, children hospitalized with RSV bronchiolitis were younger and had less previously known risk factors for RSV such as prematurity, chronic lung disease, and congenital heart disease; this finding may reflect the benefit of immunoprophylaxis against RSV. In fact, the adoption of anti-RSV prophylaxis may explain why non-RSV bronchiolitis may now be more prevalent in hospitalized children with CHD and other high-risk conditions than RSV bronchiolitis [17, 28]. Disease severity was greater in children with RSV bronchiolitis versus non-RSV bronchiolitis as measured by significant differences in length of hospitalization stay, length of stay in the PICU, intubation duration, and percentage of children requiring supplemental oxygen [7]. 

### 2.3. Cardiac Complications of RSV

 Although perhaps not as well appreciated as the pulmonary manifestations of RSV infection, the virus can lead to cardiac complications in hospitalized children [29].  Table 1 lists the cardiovascular complications that have been associated with RSV infection. In a study of otherwise healthy 69 children hospitalized with bronchiolitis in Italy, none of whom required admission to the intensive care unit or treatment with bronchodilators, sinoatrial blocks were significantly more likely to occur in RSV-positive patients than in RSV-negative patients. Fortunately, these sinoatrial blocks were always asymptomatic and 24-hour Holter ECG monitoring demonstrated complete regression of the sinoatrial blocks at  28 ± 3  days. This suggests that direct cardiac involvement can occur from RSV infection rather than secondary to lung alterations alone [30]. A study of 34 mechanically ventilated children with RSV bronchiolitis who did not have CHD reported that 20% had reduced right ventricular function and cardiac troponins were elevated in 41%. However, in this same study, there was no evidence of left ventricular dysfunction nor was there any relationship to elevations in myocardial or hepatic levels of troponin or transaminases, respectively, making it difficult to specifically attribute RSV as the cause of cardiac findings [31].

### 2.4. Treatment of Bronchiolitis

 Historically the treatment of bronchiolitis has been supportive. Maintaining adequate oxygen saturation and preventing dehydration has been the mainstay of treatment for several decades. An overview of bronchiolitis treatment in various clinical settings can be found in Table 2. In outpatients with bronchiolitis, nebulized epinephrine reduced the likelihood of hospitalization and improved clinical severity scores at 60 and 120 minutes after treatment when compared to placebo. In the inpatient setting, the only intervention that has been shown to lead to clinically meaningful outcomes such as a reduction in length of stay was nebulized hypertonic saline (3%). Chest physiotherapy demonstrates some improvement in clinical score at 1–3 days but the evidence of benefit is weak. Although there is not a recommended strategy for patients in an ICU setting, surfactant and immunoglobulin therapies along with standardized ventilation protocols may hold promise in the future [32]. It should be emphasized that the successful treatment options presented in Table 2 have excluded children with CHD, so caution is warranted when attempting to form more generalized conclusions regarding the effectiveness of these therapeutic options. Rates of bacterial pneumonia can be 20% or higher in patients with RSV infection and respiratory failure. Therefore it appears reasonable to start empiric antibiotics for 24 to 48 hours pending culture results in this group of patients [33]. 

 A common clinical scenario that health care providers face is how to evaluate and treat the presence of fever in infants with bronchiolitis, particularly those under the age of three months. Fortunately, the overall frequency of serious bacterial infections (SBIs) in a study of over 4000 patients <2 years of old, hospitalized with bronchiolitis, was low. The overall frequency of SBIs was 1.0% in RSV-positive patients and 0.7% in RSV-negative patients (not statistically significant). The most common SBI was a urinary tract infection, and there were no cases of positive cerebrospinal fluid cultures [7]. 

 Currently, respiratory support interventions for patients with severe bronchiolitis are being examined to reduce the need for mechanical ventilation but the choice of modalities is still largely based on clinical judgment and larger clinical trials are needed to define optimal strategies. In this regard, a recent retrospective study that examined heated, humidified, high-flow nasal cannula (HFNC) demonstrated a significant 68% reduction in the need for intubation in infants with bronchiolitis. HFNC allows the delivery of high inspired gas flows, with or without an increased oxygen concentration, and provides a variable degree of continuous positive airway pressure that leads to decreased work of breathing. However, prospective randomized trials are needed [34].

## 3. Immunoprophylaxis

### 3.1. Palivizumab

 Palivizumab is a humanized murine monoclonal antibody directed against the fusion protein of RSV that in preclinical trials was able to neutralize both subtypes of RSV strains and was 50- to 100-fold more potent than respiratory syncytial virus immune globulin administered intravenously (RSV-IGIV) [35]. The use of RSV-IVIG, containing high levels of RSV-neutralizing antibody, became contraindicated in the use of children with cyanotic CHD due to an increased frequency of cyanotic events. This increase in frequency of cyanotic episodes may relate to increased blood viscosity, secondary to reactive polycythemia, commonly seen in patients with a right-to-left cardiac shunt [36]. 

In a multinational, randomized, double-blind, placebo-controlled trial, children ≤24 months of age who had hemodynamically significant congenital heart disease were administered either 15 mg/kg of palivizumab or an identically appearing placebo every 30 days for a total of 5 doses during four consecutive RSV seasons (1998–2002). Palivizumab recipients experienced a significant 45% relative reduction in RSV hospitalizations when compared to placebo (9.7% hospitalization rate in placebo group vs. 5.3% in palivizumab group). Palivizumab recipients also spent significantly less days in the hospital and had less days requiring oxygen supplementation. There was also a trend for decreased need of intensive care and mechanical ventilation. The mortality rate was 3.3% in palivizumab recipients and 4.2% in placebo recipients but this did not reach statistical significance. An important clinical finding in this trial was that palivizumab serum concentrations were reduced 58% after cardiopulmonary bypass and therefore led to the recommendation that another 15 mg/kg dose of palivizumab should be administered after surgery [9].  Table 3 highlights studies that have reported on the impact of palivizumab prophylaxis in preventing RSV hospitalization rate in children with CHD.

Table 4 highlights recommendations from the American Academy of Pediatrics for palivizumab administration in children with CHD. Adherence to the prophylaxis schedule is critical since children with CHD who receive adequate RSV prophylaxis are less likely to be hospitalized with an RSV infection. López et al. reported that children who received adequate RSV prophylaxis were 58% less likely to be hospitalized than those that did not have adequate prophylaxis (3.3% vs. 7.9%) [5]. 

Children do not necessarily need to receive their first dose of palivizumab prophylaxis in the hospital setting but can reliably receive it in an office-based or home-based setting [42]. A recent systematic review of compliance with palivizumab prophylaxis reported that home-based programs are associated with improved compliance compared to office-based administration [43].

### 3.2. Motavizumab

 Motavizumab was developed by affinity maturation of palivizumab, binds to the RSV fusion protein 70-fold better than palivizumab, and reduces pulmonary RSV titers up to 100-fold lower compared to palivizumab in cotton rats [44]. 

 A multinational Phase 2 randomized, double-blind, palivizumab-controlled study of children ≤24 months enrolled 1236 patients with hemodynamically significant CHD, or acyanotic CHD with pulmonary hypertension and/or use of daily medication for congestive heart failure. The safety profiles of motavizumab and palivizumab were similar except motavizumab recipients had a significantly higher incidents of skin events such as cutaneous hypersensitivity (1.6% vs. 0.3%, resp.; *P* = 0.038).

 Although the study was not sufficiently powered to compare efficacy between the two drugs, there was a nonsignificant difference in RSV-related hospitalizations between motavizumab and palivizumab (1.9% vs. 2.6%, resp.). There was no significant difference between groups related to percentage of hospitalized patients requiring supplemental oxygen, number of intensive care unit admissions, need for mechanical ventilation, and the length of hospital stay. There was also no significant difference in medically attended lower respiratory tract infections between motavizumab and palivizumab (1.0% vs. 1.9%, resp.). Mortality rates were also similar between motavizumab and palivizumab recipients (1.5% vs. 1.6%, resp.) [45]. Because RSV replication depends on an RNA polymerase that can have a high mutation rate, there has been concern that immunoprophylaxis may increase the potential of resistant RSV mutants. Although resistant RSV variants were detected in ~5% of breakthrough cases, these did not appear to worsen the patient's clinical outcome [46]. 

 In patients who underwent cardiopulmonary bypass surgery, those who received an extra dose of motavizumab achieved a similar serum trough concentrations as those who did not undergo cardiopulmonary bypass, highlighting the need to redose motavizumab after cardiopulmonary bypass surgery. However, because of motavizumab's lack of improved efficacy over palivizumab and higher number of allergic reactions, it was not approved by the United States Food and Drug Administration and is not available for immunoprophylaxis against RSV.

### 3.3. Economic Considerations of Immunoprophylaxis

 Although immunoprophylaxis has been showed to reduce hospital admissions related to RSV, there has not been evidence that mortality has improved. As a result, controversy regarding the cost-benefit of administering palivizumab to all children <24 months of age with hemodynamically significant congenital heart disease has been raised [47], especially since the RSV hospitalization rate in children with CHD is lower in children >12 months of age [5, 19, 48]. Cost-effectiveness studies of palivizumab vary greatly in their methodology leading to disparate conclusions regarding their effectiveness. The cost-benefit of immunoprophylaxis is influenced by the rate of RSV hospitalization so that immunoprophylaxis given to an infant in the Canadian Inuit region, which has the highest rate of RSV infection globally, would lead to a different conclusion regarding cost-effectiveness than an infant where the RSV hospitalization rate is lower such as in Florida [49, 50]. In addition, how costs are assigned in economic modeling may have an impact on the cost-effectiveness of palivizumab since lost productivity and the assumption that palivizumab can reduce health-care resource utilization for recurrent wheezing have been included in some analyses [51]. 

 Although pediatric cardiologists from Europe have published similar recommendations as the AAP regarding indications for palivizumab prophylaxis [52], other countries like the UK and Switzerland have adopted a more strict policy regarding palivizumab and recommend its administration only to infants less than six months of age who have left-to-right shunt hemodynamically significant congenital heart disease and/or pulmonary hypertension (UK), or who are <12 months of age and have additional risk factors like cyanotic CHD, pulmonary hypertension, or congestive heart failure (Switzerland) [53]. 

 There has been robust discussion as to whether infection with RSV or other viruses in infancy can lead to the development of asthma since previous studies have reported that persistent respiratory symptoms and impaired lung function can occur until later childhood and even young adulthood [54, 55]. Whether viral respiratory tract infections are causal factors of asthma or merely are coincidental in a child predisposed to asthma is still unresolved [56]. Recently, a multicenter trial in Europe and Canada demonstrated that palivizumab administration given in the first year of life to premature infants significantly decreased the likelihood of recurrent wheezing in children up to 5 years of age but did not have any effect on infants who had family history of atopy, suggesting that the cause of wheezing is different between asthma and RSV [57]. 

 The debate about the cost-effectiveness about palivizumab only serves to highlight the need for more effective prevention strategies for such a prominent illness that contributes significantly to worldwide childhood morbidity and mortality.

## 4. Future Directions

 Previous attempts at developing a vaccine to prevent RSV infection have been unsuccessful due to several factors such as the early age of infection, the ability of the virus to evade preexisting immune responses, a failure of natural infection to confer immunity throughout an individual's life, a legacy of vaccine-enhanced disease, and animal models that do not precisely mimic human RSV [58]. In the 1960s, recipients of a formalin-inactivated vaccine experienced increased morbidity and mortality upon subsequent exposure to RSV [59, 60]. 

 Since this time, subunit vaccines that target the F and G proteins of RSV, the delivery of vaccine antigens by live viral vectors such as recombinant attenuated parainfluenza virus type-3 expressing RSV-F protein, and live-attenuated vaccines have undergone testing in preclinical trials and Phase I trials and may hold promise as future vaccine candidates against RSV [58, 61, 62]. Inducing neutralizing antibody to reduce antigen accumulation and inducing CD8+ T cells to clear RSV-infected cells are thought to be important principles for RSV vaccine development [58]. However, even if a vaccine emerges, it is likely that multiple doses will be needed at frequent intervals so that adequate immunity against RSV develops. This may complicate efforts to achieve the full benefits of vaccination in low-income countries where the burden of RSV is greatest. Additionally, a small interfering RNA (siRNA) that impacts RSV replication, including RNA polymerase function, was delivered via nasal spray to 88 subjects before and after they were given an inoculum of wild-type RSV and significant antiviral activity with favorable safety profile was demonstrated supporting further investigation of this approach as a treatment for RSV infection [63]. This research may also lead to the development of other locally delivered siRNAs against other respiratory pathogens.

## Figures and Tables

**Table 1:** Cardiovascular complications associated with RSV infection.

Sinoatrial block
Tachyarrhythmias
Atrioventricular block
Pericarditis
Myocarditis
Complete heart block
Reduced right ventricular function

**Table 2:** Evidence-based treatment options for bronchiolitis.

Outpatient	Inpatient	Intensive Care Unit	Not effective
Nebulized Epinephrine	Nebulized	No specific recommendations	Bronchodilators
	Hypertonic	Consider empiric antibiotics for infants with respiratory failure	Glucocorticoids
	Saline (3%)		

Source: Bialy et al. [32] and Levin et al. [33].

**Table 3:** Palivizumab prophylaxis in children with CHD and its impact on RSV hospitalization rate.

Author (reference)	Location	Methodology	Hospitalization rate without prophylaxis	Hospitalization rate with prophylaxis	Relative reduction
Feltes et al. [9]	International	Randomized, double-blind, placebo-controlled	9.7%	5.3%	45.4%*
Cohen et al. [37]	USA	Prospective, observational	N/A	1.9%	N/A
Chang and Chen [38]	California, USA	Observational	0.56%	0.46%	17.9%
Saji et al. [39]	Japan	Questionnaire	N/A	4.6%	N/A
Saji et al. [40]	Japan	Questionnaire	4.42%	2.53%	42.8%

*denotes statistical significance.

**Table 4:** Recommendations for palivizumab prophylaxis in children with CHD.

(i) ≤24 months of age with hemodynamically significant cyanotic CHD	
(a) Infants receiving medication to control congestive heart failure	
(b) Infants with moderate-to-sever pulmonary hypertension	
(c) Infants with cyanotic heart disease	
(ii) Initiate 15 mg/kg of palivizumab prophylaxis monthly during RSV season (typically November through March in North America)	
(iii) Give additional dose of palivizumab 15 mg/kg in patients who require cardiopulmonary bypass for a surgical procedure and will continue to need prophylaxis as soon as they are medically stable	

Source: [41].
